# Characterization and structural analysis of wild type and a non-abscission mutant at the development funiculus (*Def*) locus in *Pisum sativum *L

**DOI:** 10.1186/1471-2229-9-76

**Published:** 2009-06-23

**Authors:** Kwadwo Owusu Ayeh, YeonKyeong Lee, Mike J Ambrose, Anne Kathrine Hvoslef-Eide

**Affiliations:** 1 Department of Plant and Environmental Sciences, Norwegian University of Life Sciences, PO BOX 5003, 1432 Aas, Norway; 2 Department of Crops Genetics, John Innes Centre, Norwich Research Park, Colney Lane, NR4 7UH Norwich, UK

## Abstract

**Background:**

In pea seeds (*Pisum sativum *L.), the *Def *locus defines an abscission event where the seed separates from the funicle through the intervening hilum region at maturity. A spontaneous mutation at this locus results in the seed failing to abscise from the funicle as occurs in wild type peas. In this work, structural differences between wild type peas that developed a distinct abscission zone (AZ) between the funicle and the seed coat and non-abscission *def *mutant were characterized.

**Results:**

A clear abscission event was observed in wild type pea seeds that were associated with a distinct double palisade layers at the junction between the seed coat and funicle. Generally, mature seeds fully developed an AZ, which was not present in young wild type seeds. The AZ was formed exactly below the counter palisade layer. In contrast, the palisade layers at the junction of the seed coat and funicle were completely absent in the *def *mutant pea seeds and the cells in this region were seen to be extensions of surrounding parenchymatous cells.

**Conclusion:**

The *Def *wild type developed a distinct AZ associated with palisade layer and counterpalisade layer at the junction of the seed coat and funicle while the *def *mutant pea seed showed non-abscission and an absence of the double palisade layers in the same region. We conclude that the presence of the double palisade layer in the hilum of the wild type pea seeds plays an important structural role in AZ formation by delimiting the specific region between the seed coat and the funicle and may play a structural role in the AZ formation and subsequent detachment of the seed from the funicle.

## Background

Abscission is the controlled removal of a plant organ from the main plant body [1,2]. In some cases, abscission occurs at an early stage of development, a phenomenon that can be described as premature abscission. The abscission process may be an adaptive strategy of the main plant body in response to environmental stress such as temperature, disease, water, light quality and nutrition which adversely affect the parent plant body [1]. In pepper (*Capsicum annuum *L.), Gonzalez-Dugo *et al*. [3] suggested that high temperatures may be the reason for flower abscission whereas fruit abscission was reported during cold temperatures in *Lonicera maacki *[4]. In pea (*Pisum sativum *L.), high temperatures have been suggested as disrupting the development of reproductive organs leading to their abscission [5]. It has also been reported that some plants undergo floral and fruit abscission ostensibly to remove organs from the plant so that competition for pollinators and carbon assimilates are reduced [6,7]. In addition, endogenous factors such as phytohormones, auxin and ethylene and more importantly the disruptive role by either ethylene on auxin or vice versa, may play a key regulatory function in abscission [8-10].

Abscission occurs in predestined areas or positions on the plant and are referred to as abscission zones (AZ) [11,12]. The AZ is made up of multicellular structures which are morphologically distinct from surrounding cells and are formed in a few or up to several cell layers [9,13]. For example, the AZ in leaflets of *Sambucus nigra *is made up of 20–30 cell layers [14]. The cells in the AZ become larger and this is followed by dissolution of the middle lamella. The process occurs through the action of hydrolytic enzymes such as polygalacturonase [15-18] and β-endo-glucanase [19-21]. These hydrolytic enzymes are believed to dissolve the middle lamella, which function by cementing neighboring cells together, resulting in cell separation processes [22].

Abscission is of crucial importance in both agriculture and horticulture. When fruits and seeds undergo abscission, they provide an efficient and effective means of dispersal and propagation so that plants are maintained from generation to generation. However, premature abscission may result in loss of yield. The identification and manipulation of traits and processes that influence fruit and seed dispersal are therefore of great interest in the development of strategies for crop improvement through the reduction of yield losses [23]. The yield and harvestability of many agronomically important crop species have been greatly improved through selection and breeding for reduced shattering [24,25].

Mutants with altered phenotypic appearance compared to the wild type, may provide valuable insights into elucidating and understanding the biochemical and structural basis of the abscission process [26]. Such mutants have been described and characterized in a wide range of plant species. In *Arabidopsis*, the *Inflorescence Deficient in Abscission *(*IDA*) gene has been implicated in causing the petals to remain on the main plant body without being shed [27]. The *Never ripe *tomato fails to undergo many processes associated with normal fruit ripening, including abscission [28-30]. Similarly, the *jointless *mutant of tomato fails to form abscission zones at pedicel midpoints as compared to wild type plants [31-33]. The *Abs*^- ^mutant in *Lupinus angustifolius *cv. 'Danja' fails to abscise any organs despite an apparently normal pattern of growth and senescence [26]. In *Arabidopsis*, mutants *dab1-1*, *dab2-1*, *dab3-1*, *dab3-2 *and *dab3-3 *have all been shown to delay the abscission of floral parts [2] and an abscissionless leaf variety of pubescent birch has also been described [34].

Peas are one of the world's most important grain legumes and serve as a valuable protein source in the diet of humans and animals. According to the *Bi-weekly Bulletin*, *Agriculture and Agri-Food Canada *[35], dry pea production in the world has ranged between 12.5 million tones (Mt) in 1998–1999 to 9.9 Mt in 2002–2004 with France, Canada and the USA being the leading production countries. Abscission of pea seeds from the funicle helps ensure effective dispersal of seeds for food and cultivation. Significant loss of seeds however, can result from seed falling out of mature pods after heavy late season rains followed by high temperatures and dry winds which can cause the pods to split and open. While this is a relatively infrequent occurrence, loss in marginal growing regions has stimulated the evaluation of a mutation at the *def *locus into breeding programmes and a limited number of released varieties. The spontaneous *def *mutation in pea was first described by Rozental [36]. Original testcrosses revealed a simple monogenic recessive inheritance and the name and gene symbol (*Def*) for the locus of *development funiculus *[37-39]. The locus has been found to be located on the bottom end of linkage group VII corresponding to chromosome no. 4 [40-42]. Recently, von Stackelberg [43] used molecular marker techniques to map the *def *locus. However, detailed information on the structural basis of the *def *mutant has remained scarce.

In this study, structural analyses were employed to further characterize the non-abscission mutant (*def*) in two lines carrying the mutant allele and two lines carry the wild type (*Def*) allele.

## Results

### Seed abscission in wild type and *def* mutant pea

**Figure 1:**
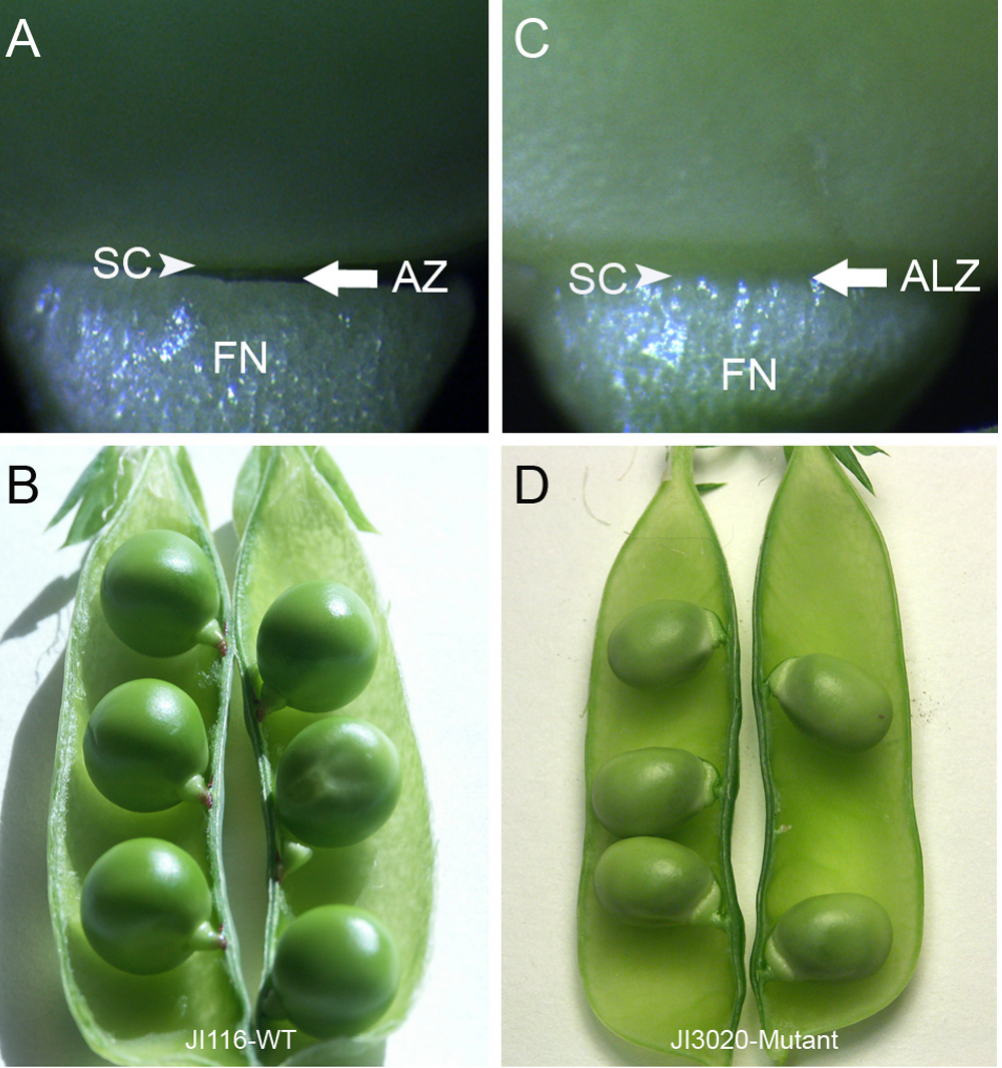
**Abscission zone (AZ) development in seeds of wild type pea JI 116 (A-B) and *def *mutant pea JI 3020 (C-D)**. (A). Distinct AZ development between funicle and seeds of the wild type pea. (B). Arrangement of pea seeds to the replum in a pod of the wild type pea. (C). Inseparable attachment of the seed to the funicle in the mutant pea. The intervening space which delimits the funicle from the seed is defined as the Abscissionless zone (ALZ). (D). Arrangement and attachment of pea seeds to the replum in a pod of the *def *mutant pea. The *def *mutant pea shows a swollen and thick funicle compared to the wild type. Arrows indicate the AZ and ALZ in the wild type and mutant, respectively; Arrow heads indicate seed coat; SC, Seed coat; AZ, Abscission zone; ALZ, Abscissionless zone

Phenotypic differences between seeds and pods of JI 116 (wild type *Def*) and JI 3020 (*def *mutant) were examined at different stages of development. In mature wild type pods, seed detachment normally occurs through the separation of the seed body from the funicle at a site referred to as the abscission zone (AZ) (Figure 1A). The distal end of the funicle (the end attached to pod wall) in wild type, does not become detached from the pod wall (Figure 1B). In the *def *mutant, the funicle was found to be accreted (strongly attached) to the seed at the same intervening hilum region which can be described as an abscissionless zone (ALZ) (Figure 1C). In contrast to the wild type, seeds of the mutant had a slightly thickened funicle. Furthermore both the proximal and distal ends of the funicle remain firmly attached to the seed coat and the pod wall, respectively (Figure 1D).

### Structural comparison of the seed/funicle interface in wild type and def mutant pea seeds

**Figure 2:**
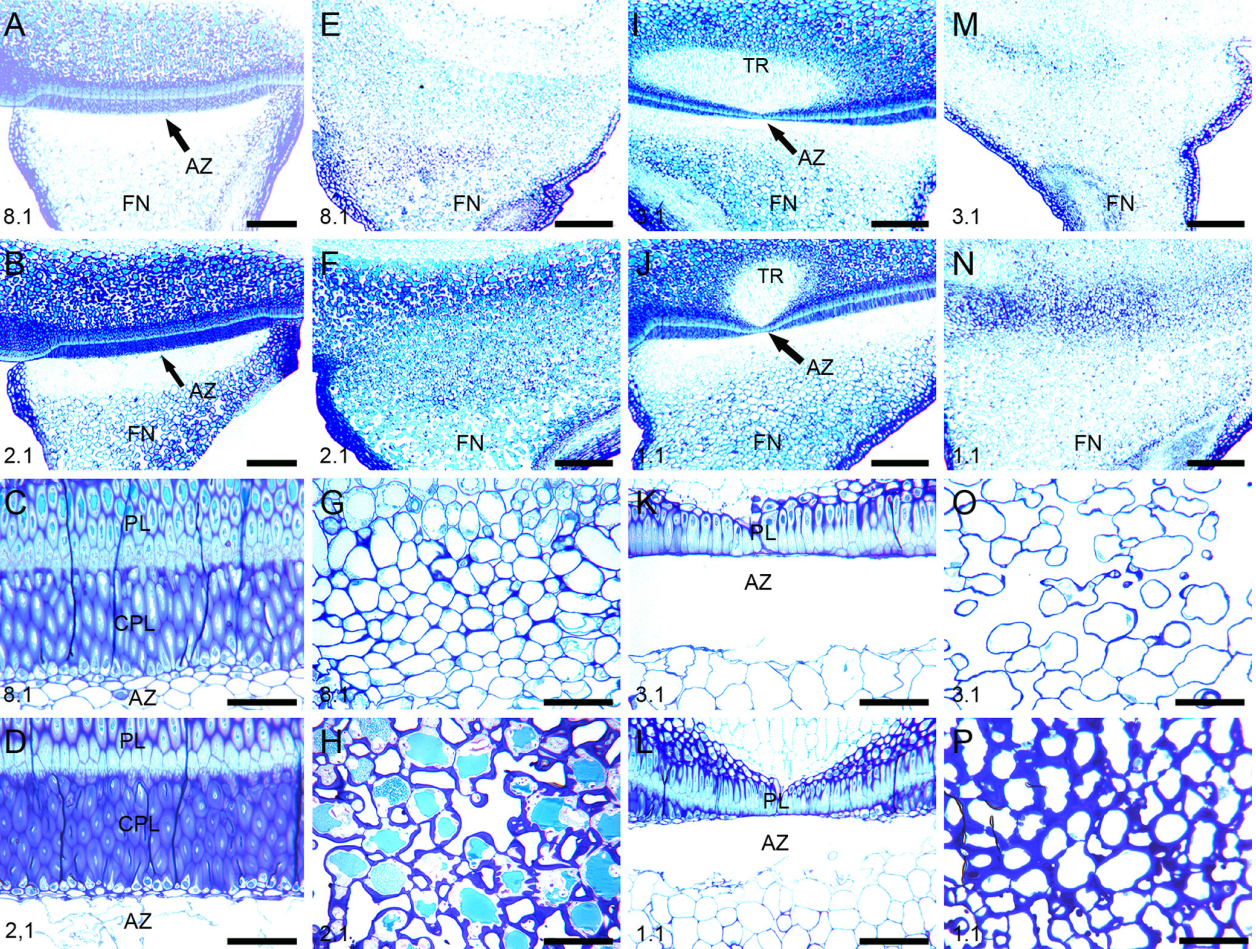
**Light micrographs showing structural differences between two wild types and two *def *mutant pea lines**. (A-D). The wild type (JI 116). (A) AZ development in young pea seed at stage 8.1 and (B) In mature pea seed at 2.1. (C) Higher magnification of the AZ development in the young pea seed in (A). (D) Higher magnification of the AZ in the mature pea seed in (B). There is no sign of cell separation in young stage at 8.1 but distinct cell separation occurs in the mature stage at 2.1. (E-H) The *def *mutant type (JI 1184). (E) Non-abscission in young mutant pea seed at stage 8.1 showing the absence of the hilum palisade layer. (F) Non-abscission in mature mutant seed at stage 2.1. (G-H) Higher magnifications of the abscissionless zones (ALZ) in young and mature seeds of the *def *mutant in E and F, respectively. (I-L) The wild type (JI 2822). (I) AZ development in the wild type pea at stage 3.1. (J) AZ development in the mature pea at stage 1.1. (K-L) Higher magnification of the AZ in (I) and (J), respectively. (M-P) The *def *mutant type (JI 3020). (M) Non-abscission in young mutant pea seed at stage 3.1. (N) Mutant pea seed at stage 1.1. (O-P) Higher magnification of the ALZ in (M) and (N), respectively. Seeds in the first (most mature) pod and close to pea stock are designated as 1.1. The youngest pod and close to the pea stock is designated as 8.1 for JI 116, 8.1 for JI 1184, 3.1 for JI 2822 and 3.1 for JI 3020. AZ, Abscission zone; FN, Funicle; PL, Palisade layer; CPL, Counter palisade layer; TR, Tracheid bar. Scale bars = A, B, E, F, I, J, M and N = 12.5 μm; C, D, G, H, K, L, O and P = 25 μm.

A structural comparison between wild type and *def *mutant pea seeds revealed that both the wild type lines (JI 116 and JI 2822) exhibited a distinct double palisade layer in the hilum region which served to define the AZ (Figure 2A–D, I–L). The layer proximal to the seed, is described as the palisade layer whereas an opposing palisade layer is described as the counter palisade layer (Figure 2C and 2D). In young wild type seeds, cell separation was not observed (Figure 2A and 2C), the cells remaining intact and of regular round and compact form (Figure 2C). In maturing seeds, cell separation in the AZ occurred immediately below the counter palisade layer in the hilum region (Figure 2B) with cell separation starting in the middle and developed outwards to the epidermis of the funicle. These cells were characterized as being irregular and damaged (Figure 2D). In wild type JI 2822, the abscission process was again observed in seeds that were well into their maturation phase, at and around the time of maximum fresh weight and started at the midpoint where the counterpalisade layer was inconspicuous (Figure 2I–L). The same sequence of cell separation was observed in the wild type line JI116 with the cell separation process starting in the centre and extending outwards towards the epidermis of the AZ and the seed finally becoming separated from the funicle. In contrast, seeds of the mutant pea lines did not develop a distinct boundary region of a double palisade layer between the seed coat and the funicle (Figure 2E–F and Figure 2M–N). Moreover, no cell separation events were observed even in mature pea seed (Figure 2F and 2N) thus the funicle remained firmly bound to the seed (Figure 2G–H and Figure 2O–P).

## Discussion

### Abscission of seeds in wild type and mutant is controlled by *Def* loci

The abscission process is defined as the shedding of organ parts such as leaves, flowers and fruits [12]. Our study focused on a structural comparison between the wild type and *def *mutant pea seed. These two pea types exhibited distinctively different phenotype and structural differences with respect to the region where the funicle abuts the hilum. The *Def *wild type lines underwent a normal abscission event between funicle and seed coat mediated by cell separation in a specific layer of cells immediately below the counter palisade layer. No abscission event occurred in the *def *mutant lines which lacked the double palisade in the hilum region. We conclude, therefore, that the *Def *locus is important in controlling the abscission event of pea seeds.

### Absence of the hilum palisade layers is the key characteristic in the *def* mutant pea seed

Structural analysis revealed the absence of the palisade layer and counterpalisade layer underlying the funicle in *def *mutant pea seeds whereas the wild type showed a distinct double palisade layers at the same location. In the testas of wild type pea seeds, the palisade layers in the hilum take their origin from the outer integuments and are made up of macrosclereids [44] which are elongated perpendicular to the surface of the seed [45,46]. The testas of the mutant lines are similarly covered by a layer of macrosclereids, but this is not continued into funicle region which lacks any palisade structures. Although there is no direct evidence that the double palisade layer underlying the funicle is responsible for the abscission of seed from the funicle in the wild type pea, the absence of the double palisade layers in the non-abscission *def *mutant pea suggest that the palisade layers may play a key role in regulating the abscission process in some way.

The palisade layers in seeds are also responsible for water permeability. In seed development, seed maturation is accompanied with reducing moisture content in the seed [47]. The testa comprises of a layer of strengthened palisade cells and these cells which are implicated in controlling permeability both during development and at final maturity [48]. *def *mutant peas develop normal testas therefore the mutant is clearly not defective in making cells analogous to palisade cells that are normally found in the hilum region. Further study is necessary to probe the regulatory basis of the failure to develop the palisade layers underlying the funicle in *def *mutant seeds which would otherwise go on to develop an abscission event in wild type seeds.

### Cell separation process in the AZ of wild type seed

We have shown that the abscission of the seed from the funicle is initiated at the centre of the seed coat/palisade junction in the wild type line (JI 116) (Figure 2B and 2D). In JI 2822, the abscission event was also observed to start at the centre of the seed coat/palisade junction, particularly where the counterpalisade layer becomes restricted in the vicinity of the tracheid bar (Figures 2I and 2J). Although it is not shown, the other wild type and both mutant lines also possessed tracheid bars. The *def *mutant seeds were clearly able to develop and mature as fully functional seeds and the loss of the double palisade layer and failure to develop an AZ were not critical to their development.

The actual separation of cells in the AZ begins in the single layer of cells directly beneath the counter palisade layer and extends outwards as abscission proceeds. This is in contrast to poinsettia flower abscission, where active cell division and cell enlargement occur during the abscission process [49]. However, such cell divisions are not a prerequisite prior to abscission as in tobacco, tomato and several other solanaceous genera [50]. Like pea, these plants have a visible AZ long before abscission is initiated and do not shows cell expansion. Cell swelling has been suggested as assisting in breaking of the vascular strand [49,51]. In our study, it was hard to see cell expansion or cell swelling in the AZ as cells in the AZ were in very irregular conformation and cell walls frequently appeared damaged and broken. Although no enzyme assay was performed in this study, it is plausible to suggest that cells in the AZ may have been attacked by hydrolytic enzymes. This is especially the case where cell separation is accompanied with cell wall modification where the cell wall components disappear or are reconstruct. Many studies on enzyme activity during abscission have been focused on enzymes that provide cell wall dissolution [20,21,52]. Expression of such enzymes are dependent on maturity, leading to the dissolution of the middle lamella between adjacent cells [53]. The identification and localization of such enzymes in further studies into pea seed abscission offer a further role of the *def *mutant in helping to understand the cellular context in which genes that encode for such enzymes are transcribed and expressed.

## Conclusion

This study provides a structural comparison of the distinct double palisade layer and the AZ found in the hilum region of wild type pea seeds and the absence of the double palisade and non abscission lines carrying the *def *mutant allele. These findings underline key regulation of the *Def *locus in controlling the abscission process through the correct development of the hilum double palisade layer as a prerequisite for AZ development in wild type pea seeds.

## Methods

### Plant material

The four lines of pea (*Pisum sativum *L.) seeds JI 116, JI 2822, JI 1184 and JI 3020 used in this study were selected on the basis of the presence of specific alleles at the *Def *locus, which control the detachment of the seed from the funicle (Table 1). JI 1184 originates from Rozenthal's collection from Russia where the *def *mutation was first identified and isolated and is an early line selected as carrying the *def *allele. It has been used for agronomic studies and is a sister line to the type line for *def *mutant allele. JI 3020 is a registered cultivar from the Netherlands that incorporates the same mutant *def *allele. In the absence of near-isogenic lines for the *Def *alleles, two well characterized lines (JI 116 and JI 2822) that matched the gross plant habit of the mutant lines were selected. Both these lines are well characterized genetically and were selected for use in genetic analysis of heterozygous *Def/def *seeds that are the subject of further study of this locus.

**Table 1:** Details of *Pisum sativum *accessions and their allelic status with respect to the *Def *locus.

Accession	Name	*Def *allele
JI 116	cv. Parvus	*Def (*wild type)

JI 2822	RIL, research line	*Def (*wild type)

JI 1184	Priekuskij-341-def	*def (*mutant)

JI 3020	cv. Nord	*def (*mutant)

Seeds corresponding to each line were sown in pots with fertilised peat (Floralux, Nittedal Torvindustrier, Norway) and grown under greenhouse conditions at 22°C and 16/8 h photoperiod with a photon flux of 110 μmol m^-2 ^s^-1 ^(400–700 nm Phosynthetic Active Radiation (PAR)) and a daylength extending light provided from incandescent lamps (OSRAM, Germany). Seeds and seedlings were watered six days a week and given a complete nutrient solution once a week.

### Plant tissue preparation and examination

For structural analysis, seeds of all lines were embedded in LR White resin (London Resin Company, England). Seeds from each pod identification stage were transversely cut into 2 mm thick, from the funicle-seed coat interface. The cut material was further longitudinally cut into two pieces and immediately fixed in 1% formaldehyde, 0.025% glutaraldehyde, 0.1% (v/v) Tween 20 in 0.01 M sodium phosphate buffer, pH 7.2 and vacuum infiltrated for 1 h. Fixed and infiltrated tissues were placed at 4°C overnight. The fixed samples were washed twice with sodium phosphate buffer for 4 h. Washed samples were then dehydrated in a graded ethanol series. Infiltration was performed with a progressively increasing ratio of LR white resin to ethanol. At the end of the infiltration process, the specimens were transferred to an embedding mould and polymerised at 50°C for 24 h. Plant materials embedded in LR white blocks were sectioned with a diamond knife (Diatome Ltd., Switzerland) on an ultramicrotome (Leica, Germany). Sections (1 μm thick) were placed on Vectabond (Vector Laboratories, USA) coated glass slides and heated at 55°C on a warm plate to adhere the sections to the slide. For histological staining, sectioned materials were stained with toluidine blue O (Sigma, USA), washed with distilled water and mounted in Depex (BDH, USA). Sections were examined using a Leica brightfield microscope (Leica, Germany).

## Authors' contributions

KOA contributed to the growing of the plants, harvested materials, carried out the structural examination and drafted the manuscript. YKL participated in designing the experiments, structural analysis and the drafting of the manuscript. MA contributed with plant material, the general idea of the study and participated in revision of the manuscript. AKHE participated in the general idea of the study, the design of the experiments and contributed to the writing and revision of the paper. All authors have read and approved the final manuscript.
